# Application of a Simplified Method for Estimating Perfusion Derived from Diffusion-Weighted MR Imaging in Glioma Grading

**DOI:** 10.3389/fnagi.2017.00432

**Published:** 2018-01-08

**Authors:** Mengqiu Cao, Shiteng Suo, Xu Han, Ke Jin, Yawen Sun, Yao Wang, Weina Ding, Jianxun Qu, Xiaohua Zhang, Yan Zhou

**Affiliations:** ^1^Department of Radiology, Renji Hospital, School of Medicine, Shanghai Jiao Tong University, Shanghai, China; ^2^Department of Neurosurgery, Renji Hospital, School of Medicine, Shanghai Jiao Tong University, Shanghai, China; ^3^GE Healthcare China, Shanghai, China

**Keywords:** glioma perfusion, glioma grading, diffusion-weighted MRI, intravoxel incoherent motion, dynamic contrast-enhanced MRI

## Abstract

**Purpose**: To evaluate the feasibility of a simplified method based on diffusion-weighted imaging (DWI) acquired with three *b*-values to measure tissue perfusion linked to microcirculation, to validate it against from perfusion-related parameters derived from intravoxel incoherent motion (IVIM) and dynamic contrast-enhanced (DCE) magnetic resonance (MR) imaging, and to investigate its utility to differentiate low- from high-grade gliomas.

**Materials and Methods**: The prospective study was approved by the local institutional review board and written informed consent was obtained from all patients. From May 2016 and May 2017, 50 patients confirmed with glioma were assessed with multi-*b*-value DWI and DCE MR imaging at 3.0 T. Besides conventional apparent diffusion coefficient (ADC_0,1000_) map, perfusion-related parametric maps for IVIM-derived perfusion fraction (*f*) and pseudodiffusion coefficient (D*), DCE MR imaging-derived pharmacokinetic metrics, including K^trans^, v_e_ and v_p_, as well as a metric named simplified perfusion fraction (SPF), were generated. Correlation between perfusion-related parameters was analyzed by using the Spearman rank correlation. All imaging parameters were compared between the low-grade (*n* = 19) and high-grade (*n* = 31) groups by using the Mann-Whitney *U* test. The diagnostic performance for tumor grading was evaluated with receiver operating characteristic (ROC) analysis.

**Results**: SPF showed strong correlation with IVIM-derived *f* and D* (*ρ* = 0.732 and 0.716, respectively; both *P* < 0.001). Compared with *f*, SPF was more correlated with DCE MR imaging-derived K^trans^ (*ρ* = 0.607; *P* < 0.001) and v_p_ (*ρ* = 0.397; *P* = 0.004). Among all parameters, SPF achieved the highest accuracy for differentiating low- from high-grade gliomas, with an area under the ROC curve value of 0.942, which was significantly higher than that of ADC_0,1000_ (*P* = 0.004). By using SPF as a discriminative index, the diagnostic sensitivity and specificity were 87.1% and 94.7%, respectively, at the optimal cut-off value of 19.26%.

**Conclusion**: The simplified method to measure tissue perfusion based on DWI by using three *b*-values may be helpful to differentiate low- from high-grade gliomas. SPF may serve as a valuable alternative to measure tumor perfusion in gliomas in a noninvasive, convenient and efficient way.

## Introduction

Cerebral gliomas are the most common type of primary malignant brain tumors and have a poor survival rate compared with other tumor types (Kelly, 2010). The grading of glioma is of great importance in determining treatment strategies and evaluating prognosis (Klein et al., 2002). It is well known that angiogenesis is an important histopathologic feature in glioma progression and increased vascularity is typically associated with higher tumor grade. Dynamic contrast-enhanced (DCE) magnetic resonance (MR) imaging has been applied in gliomas for the purpose of characterizing tumor perfusion by providing information on vascular architecture, integrity and volume (Sorensen et al., 2009; Heye et al., 2014). However, DCE MR imaging needs the use of intravenous contrast media, which is not acceptable in patients with renal dysfunction or individuals who cannot tolerate intravenous contrast injection.

Diffusion-weighted imaging (DWI), which derives its image contrast from differences in the motion of water molecules within tissues, is a noninvasive and endogenous-contrast imaging technique that has proven useful for tumor characterization and treatment response evaluation (Le Bihan, 1991; Koh and Collins, 2007). In clinical routines, apparent diffusion coefficient (ADC) is a commonly used quantitative metric calculated from DW images. However, it has been recognized that ADC value calculated by a monoexponential model is not only affected by tissue diffusion but also microcirculation in capillary network (Le Bihan et al., 1988; Koh et al., 2011). *In vivo*, tissue ADC values are often higher than expected, which is attributed to the perfusion effect from microcirculation (Le Bihan et al., 1988; Koh et al., 2011).

Intravoxel incoherent motion (IVIM) was initially proposed by Le Bihan (1991) suggesting that using a more sophisticated approach (biexponential model) to describe the relationship between signal attenuation and *b*-values would tease out perfusion from DW data. Using IVIM-based analysis, it is possible to derive quantitative parameters that reflect tissue diffusion and perfusion separately: pure diffusion coefficient (D), perfusion-related pseudodiffusion coefficient (D*) and perfusion fraction (*f*). In normal perfused tissue at low *b*-values (<200 s/mm^2^), perfusion has a significant contribution to the DW signal, while at high *b*-values (>200 s/mm^2^), pure diffusion accounts for a large portion of the measured signal (Koh and Collins, 2007; Koh et al., 2011; Iima and Le Bihan, 2016). It is also worthwhile remembering that, although ADCs calculated from only low *b*-values (ADC_low_) are perfusion sensitive and the ADCs including high *b*-values are predominantly diffusion sensitive, the ADC_low_ is also affected by diffusion effects.

In recent years, many studies have demonstrated the usefulness of IVIM in tumor grading and differentiating recurrent tumor from treatment effect in gliomas (Klein et al., 2002; Kang et al., 2011). However, some limitations that impede more widespread adoption of IVIM should not be neglected. First, the most common clinical concern is whether there is a clear relationship between the perfusion properties derived from IVIM and other imaging techniques using intravascular tracers including DCE MR imaging and dynamic susceptibility contrast (DSC) MR imaging (Koh et al., 2011; Iima and Le Bihan, 2016). Several studies have been conducted to evaluate the relationship and inconsistent results have been reported: results from most studies showed significant correlations between IVIM and DCE/DSC MR imaging (Federau et al., 2014; Suh et al., 2014; Togao et al., 2016; Kapadia et al., 2017), whereas some studies reported no correlation (Bisdas et al., 2015; Wu et al., 2015). Second, a large number of *b*-values are needed to fully characterize biexponential signal attenuation and provide more data support for parameter estimation. Thus, eight or more *b*-values are commonly used in the published literature (Federau et al., 2014). However, this would inevitably cause a long scanning duration, which makes it less practical for clinical use.

Thus, the development of a simplified method to estimate the perfusion characteristics of gliomas might be helpful, as an alternative less time-consuming approach to IVIM and an effective perfusion-related imaging modality associated with DCE MR imaging. Therefore, the purpose of the study was to evaluate the application of a simplified method based on DWI acquired with three *b*-values to measure tissue perfusion linked to microcirculation, to validate its associations with IVIM and DCE MR imaging derived perfusion-related parameters, and to investigate its utility to differentiate low- from high-grade gliomas.

## Materials and Methods

### Patients

The protocol of this prospective single-center study was approved by the Research Ethics Committee of Renji Hospital, School of Medicine, Shanghai Jiao Tong University. All subjects gave written informed consent in accordance with the Declaration of Helsinki before their participation in the study.

Between May 2016 and May 2017, patients with suspected gliomas who underwent brain MR imaging examination and were subsequently scheduled for neurosurgical resection were considered to be included in this study. Patients were excluded if they had history of relevant biopsies or therapies before or had contraindications to MR imaging (claustrophobia, metal implants, or pacemakers). Of the 65 consecutive patients initially recruited for the study, 12 patients were excluded because of pathologic diagnosis other than gliomas according to the World Health Organization (WHO) criteria, and three patients were excluded owing to head movement artifacts. A total of 50 patients (33 men and 17 women; age range, 13–74 years; mean age, 53.2 ± 16.4 years) with pathologically confirmed gliomas were included in this study. The interval between MR imaging and the surgery was shorter than 10 days in all patients.

### MR Image Acquisition

All MR images were obtained with a 3.0-T MR imager (Signa HDxt; GE Medical Systems, Milwaukee, WI, USA) with an eight-channel head coil. Standard MR imaging protocol for brain tumors at our institution included conventional nonenhanced anatomic sequences including T1-weighted, T2-weighted, and fluid attenuated inversion recovery imaging and contrast-enhanced T1-weighted imaging. Intravenous administration of gadopentetate dimeglumine (Magnevist; Bayer Healthcare, Berlin, Germany) was performed for contrast-enhanced imaging.

DW images were acquired before the injection of contrast material. For DWI, we performed a single shot echo-planar sequence in the axial plane with the following parameters: repetition time (TR) ms/echo time (TE) ms, 3000/106; section thickness, 5 mm; intersection gap, 1 mm; field of view (FOV), 260 × 260 mm; matrix, 192 × 192; number of sections, 15; *b*-values, 0, 20, 50, 80, 150, 200, 300, 500, 800, 1000 s/mm^2^; number of signal averages, 2. Diffusion sensitizing gradients were applied sequentially in three orthogonal directions and trace images were generated. Total acquisition time for the multi-*b*-value DWI was 5 min and 36 s.

DCE MR images were obtained with a three-dimensional gradient-echo T1-weighted sequence. Pre-contrast scan with four dynamics was first performed with the following parameters: TR/TE, 3.3/1.3, flip angle, 15°; section thickness, 2 mm; FOV, 220 × 220 mm; matrix, 256 × 160; number of sections, 40. Post-contrast multiphase scan was immediately performed using identical imaging parameters in conjunction with an injection of gadopentetate dimeglumine (0.1 mmol/kg body weight) at a rate of 4 mL/s by using a power injector (Spectris; Medrad, Pittsburgh, PA, USA). Total acquisition time for DCE MR imaging was approximately 4 min.

### MR Image Analysis

#### DCE MR Image Analysis

DCE MR images were analyzed by using a commercially available software (MIStar; Apollo Medical Imaging, Melbourne, VIC, Australia). The two-compartment Tofts model was used to calculate pharmacokinetic parameters including volume transfer constant (K^trans^), extravascular extracellular volume fraction (v_e_), and vascular plasma volume fraction (v_p_; Tofts et al., 1999). Arterial input function was determined semiautomatically in intracranial internal carotid artery. Parametric maps of K^trans^, v_p_, and v_e_ were generated subsequently.

#### Diffusion-Weighted MR Image Analysis

DW MR images were processed by using an in-house program implemented with software (Matlab 2016a; MathWorks, Natick, MA, USA). The ADC value was calculated with a monoexponential model with two *b*-values (*b*_low_ and *b*_high_): *S*_high_ = *S*_low_ exp[(*b*_low_ − *b*_high_)*ADC*_low, high_], where *S*_high_ and *S*_low_ denote the DW signal intensities obtained by *b*-values of *b*_high_ and *b*_low_, respectively. By this procedure, ADC maps at low *b*-values (*ADC*_0,200_) and high *b*-values (*ADC*_200,1000_) were generated. As was previously mentioned, *ADC*_200,1000_ reflects almost only diffusion characteristics of tissues, whereas *ADC*_0,200_ indicates both diffusion and perfusion. Therefore, the difference value between *ADC*_0,200_ and *ADC*_200,1000_ can be reasonably used as a measure of tissue perfusion, which was calculated and designated as *ADC*_perf_ (*ADC*_perf_ = *ADC*_0,200_ − *ADC*_200,1000_). This assumption was confirmed by Thoeny et al. (2005) and Teruel et al. (2016), in the previously published work on rat implanted tumors and human breast tumors, respectively. According to previous studies (Federau et al., 2012; Suo et al., 2016), a cutoff *b*-value of 200 s/mm^2^ was used in the study to separate perfusion and diffusion components. We introduce here an additional parameter called simplified perfusion fraction (SPF), which represents the relative fraction of perfusion, as follows: *SPF* = *ADC*_perf_/*ADC*_0,200_. Additionally, the ADC map (*ADC*_0,1000_) used in clinical routine diagnosis was calculated with *b*-values of 0 and 1000 s/mm^2^. IVIM data analysis was performed by using a standard biexponential model: *S*_b_ = *S*_0_[*f* exp(−*b*D*) + (1 − *f*) exp(−*b*D)], where *S*_b_ is the signal intensity at a given *b*-value and *S*_0_ the signal intensity without diffusion gradient; *D* and D* are the diffusion coefficient and pseudodiffusion coefficient related to the tissue diffusivity and microvascular perfusion, respectively; *f* is the IVIM-based perfusion fraction.

#### Region of Interest Analysis

The analysis of the image data was independently performed by two neuroradiologists (MC and YZ, with 5 and 18 years of experience in neurological MR image interpretation, respectively) who were blinded to the histopathologic results. Each neuroradiologist carefully placed round-shaped regions of interest (ROI) in the solid portion of tumor on parametric maps with reference to conventional MR images by using the hot-spot method (Suh et al., 2014; Togao et al., 2016). Hot-spot ROIs were obtained by choosing the highest values on perfusion-related parametric maps (SPF, *f*, D*, K^trans^, v_p_, and v_e_) and lowest values on diffusion-related maps (*D* and ADC_0,1000_). The solid portion was defined as the enhancing region of the tumor on contrast-enhanced T1-weighted images. For the nonenhancing tumors, ROI was drawn at the center of the portion where the T2-signal abnormality was noted after careful inspection of T1- and T2-weighted images (Choi et al., 2013; Park et al., 2016). Areas of necrosis, cyst, hemorrhage, large vessels and calcifications were avoided to ensure the accuracy of the measurements. For correlation analysis of perfusion-related parameters, the tumor ROIs for SPF were propagated to the corresponding IVIM and DCE MR imaging maps.

### Statistical Analysis

Statistical analyses were performed with commercially available softwares (SPSS, version 17.0, SPSS, Chicago, IL, USA; MedCalc, version 11.4.2.0, MedCalc Software, Mariakerke, Belgium and GraphPad Prism 5, GraphPad Software, La Jolla, CA, USA). Interobserver agreement for each parameter from the two neuroradiologists was analyzed by using the intraclass correlation coefficient, where intraclass correlation coefficient values higher than 0.80 were deemed as an almost perfect level of agreement. In case of an almost perfect level of agreement, the average value of each parameter for each subject measured by two radiologists was obtained and used for further statistical analyses. Correlation between perfusion-related parameters was computed by using the nonparametric Spearman rank correlation. Correlation coefficients were interpreted as follows: very weak, 0.2; weak, 0.4; moderate, 0.6; strong, 0.8; and very strong, 1. The Mann-Whitney *U* test was used for the comparison of each parameter between low- and high-grade gliomas. Receiver operating characteristic (ROC) curves were employed to calculate the area under the curve (AUC) and to determine the optimal thresholds for grading gliomas by maximizing the Youden index. AUCs were compared by using the method developed by DeLong et al. (1988). To prevent overestimation by using the same population to construct classifiers and validate performance, a leave-one-out cross validation method was applied. Results with *P* values less than 0.05 were considered to indicate a significant difference. Bonferroni correction was applied to control for multiple comparisons where applicable.

## Results

Histopathologic examinations of surgical specimens revealed that 19 of 50 patients (38%) were confirmed with low-grade (WHO grade II) gliomas and 31 patients (62%) with high-grade (WHO grades III and IV) gliomas. Descriptive statistics regarding the demographic and histopathologic characteristics in study group are provided in Table 1. For interobserver agreement analysis, intraclass correlation coefficient values were 0.94 for SPF, 0.88 for ADC_0,1000_, 0.92, 0.89 and 0.83 for IVIM derived *f*, D and D*, and 0.89, 0.92 and 0.81 for DCE MR imaging derived K^trans^, v_e_ and v_p_.

**Table 1:** Demographic and histopathologic characteristics in study group.

Characteristic	Datum
Mean age (y)^*†^	
Low-grade glioma	44.1 (13–72)
High-grade glioma	58.5 (26–74)
Sex distribution (M/F)^‡^	
Low-grade glioma	11/8
High-grade glioma	22/9
WHO grade	
II	19
III	5
IV	26
Histologic type	
Astrocytoma	13
Oligodendroglioma	4
Oligoastrocytoma	2
Anaplastic astrocytoma	3
Anaplastic oligodendroglioma	2
Anaplastic oligoastrocytoma	1
Glioblastoma	25

Note—Data are numbers of patients unless otherwise indicated. *Data are mean values with range in parentheses. ^†^A significant difference in age was noted between low- and high-grade groups (*P* = 0.026). ^‡^No significant difference in sex distribution was noted between low- and high-grade groups (*P* = 0.373).

### Correlation between Perfusion-related Parameters from DWI and DCE MR Imaging

**Figure 1:**
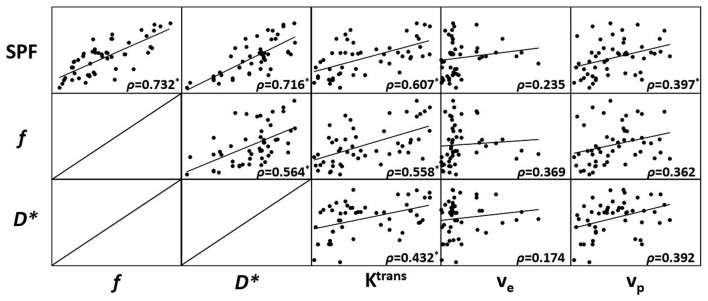
Scatterplot matrix of measured simplified perfusion fraction (SPF), intravoxel incoherent motion (IVIM) and dynamic contrast-enhanced (DCE) MR imaging parameters. Spearman rank correlation (*ρ*) was included in each scatterplot with significant correlation marked with (*). Adjusted level of significance was set at *P* = 0.004 with Bonferroni correction for 12 comparisons.

Results from Spearman rank correlation analysis between SPF, IVIM parameters and DCE MR imaging parameters are shown with a scatterplot matrix in Figure 1. There was a statistically significant strong positive correlation between SPF and *f* (*ρ* = 0.732; *P* < 0.001), and between SPF and D* (*ρ* = 0.716; *P* < 0.001). Notably, SPF exhibited a stronger correlation with K^trans^ compared with *f* (*ρ* = 0.607 and 0.558, respectively; both *P* < 0.001). Additionally, we also observed a weak correlation between SPF and v_p_ (*ρ* = 0.397; *P* = 0.004).

### Differences in DWI and DCE MR Imaging Parameters between Low- and High-grade Gliomas

**Figure 2:**
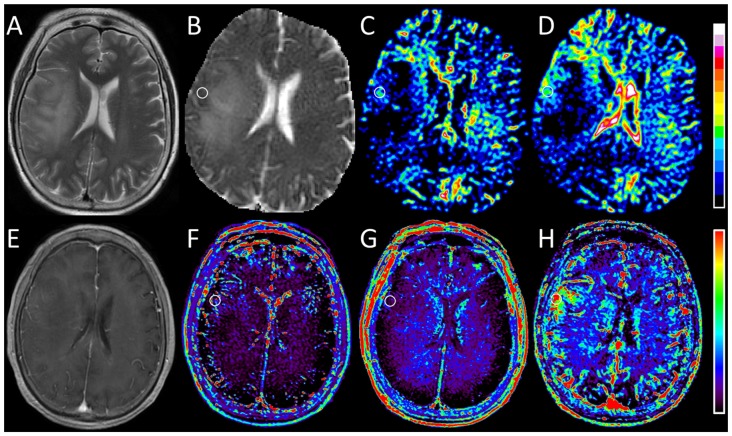
Images obtained in a 67-year-old woman with oligodendroglioma (WHO grade II). **(A)** T2-weighted image shows a hyperintense lesion with massive edema in the right hemisphere. **(B)** ADC_0,1000_ map shows increased apparent diffusion coefficient (ADC) value in the edema and slightly decreased ADC value in the corresponding area of the contrast-enhanced lesion as shown in **(E)**. **(C,D)** IVIM *f* and SPF maps show relatively increased *f* and SPF values in the corresponding low-diffusion tumoral area compared with surrounding edema. **(E)** Contrast-enhanced T1-weighted image shows faint enhancement in the tumor. **(F,G)** DCE MR imaging parametric maps of K^trans^ and v_e_ show almost isointensity in the corresponding area of the contrast-enhanced lesion. **(H)** DCE MR imaging parametric map of v_p_ shows increased v_p_ value in the corresponding area of the contrast-enhanced lesion. Note that the tumoral hyperperfusion area on v_p_ map is consistent with the region on *f* and SPF maps. Round-shaped regions of interest are marked on parametric maps.

**Figure 3:**
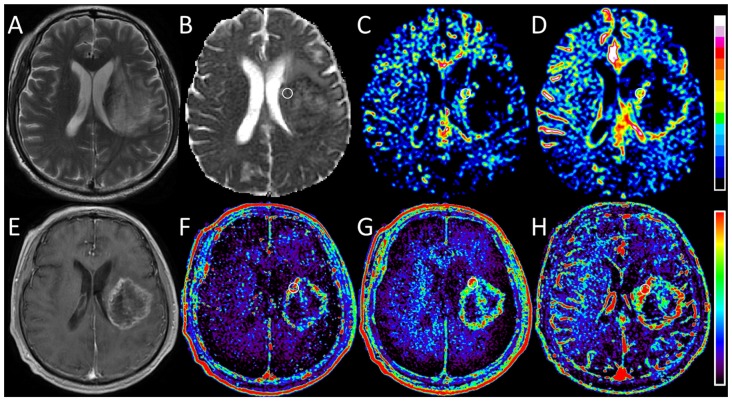
Images obtained in a 56-year-old man with glioblastoma (WHO grade IV). **(A)** T2-weighted image shows a heterogeneous hyperintense lesion in the left hemisphere. **(B)** ADC_0,1000_ map shows markedly decreased ADC value in the lesion. **(C,D)** IVIM *f* and SPF maps show increased *f* and SPF values in the corresponding area of the contrast-enhanced lesion as shown in **(E)**. **(E)** On contrast-enhanced T1-weighted image, a rim-enhanced mass with central necrotic changes is seen correspondingly. **(F–H)** DCE MR imaging parametric maps of K^trans^, v_e_ and v_p_ show markedly increased values in the corresponding area of the contrast-enhanced lesion. When focusing on *f* and SPF maps, we can notice that the tumoral hyperperfusion area on SPF map is more visually obvious and more similar to that on DCE maps. Round-shaped regions of interest are marked on parametric maps.

Statistical results from the DWI and DCE MR imaging analysis in both the low- and high-grade gliomas are reported in Table 2. Figures 2, 3 show representative cases of each patient group (WHO grades II and IV, respectively). Lower diffusivity and higher perfusion values were observed in the high-grade case compared with the low-grade case. By using the Mann-Whitney *U* test, we observed significant differences between the two groups in ADC_0,1000_ and D (*P* = 0.003 and 0.006, respectively). Perfusion-related parameters including DWI derived SPF, *f*, and D*, and DCE MR imaging derived K^trans^, v_e_ and v_p_ all exhibited significantly elevated values in high-grade gliomas than those in low-grade gliomas (all *P* < 0.05). Specifically, the median SPF in high- and low-grade gliomas was 23.56% (interquartile range [IQR], 21.73%–31.49%) and 10.47% (IQR, 7.71%–14.23%), respectively, at *P* < 0.001 for comparison.

**Table 2:** Parameters derived from diffusion-weighted imaging (DWI) and dynamic contrast-enhanced (DCE) magnetic resonance (MR) imaging between low- and high-grade gliomas.

Parameter	Low-grade glioma (*n* = 19)	High-grade glioma (*n* = 31)	*P* value
ADC_0,1000_ (×10^−3^ mm^2^/s)	1.26 (1.07–1.34)	1.03 (0.79–1.13)	0.003
SPF (%)	10.47 (7.71–14.23)	23.56 (21.73–31.49)	<0.001
*f* (%)	4.05 (3.08–5.80)	9.22 (6.83–14.41)	<0.001
D (×10^−3^ mm^2^/s)	1.22 (1.03–1.28)	1.02 (0.77–1.11)	0.006
D* (×10^–^^3^ mm^2^/s)	7.88 (4.95–10.32)	12.69 (12.09–14.77)	<0.001
K^trans^ (min^−1^)	0.041 (0.018–0.058)	0.140 (0.076–0.192)	<0.001
v_e_	0.132 (0.023–0.228)	0.204 (0.165–0.559)	0.009
v_p_	0.029 (0.020–0.058)	0.055 (0.033–0.076)	0.041

*Note—Data are medians with interquartile range in parentheses*.

### Diagnostic Performance of DWI and DCE MR Imaging Parameters for Differentiation between Low- and High-grade Gliomas

**Figure 4:**
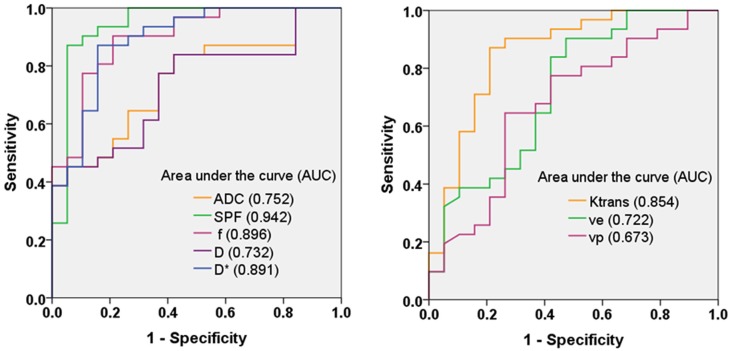
Receiver operating characteristic (ROC) curves and corresponding area under the curve values for diffusion-weighted imaging (DWI) parameters (ADC_0,1000_, SPF, *f*, D and D*) and DCE MR imaging parameters (K^trans^, v_e_ and v_p_) in the differentiation of low- and high-grade gliomas. SPF showed the highest diagnostic performance with the area under the curve value of 0.942.

Figure 4 shows ROC curves and corresponding AUCs of each parameter for differentiation between low- and high-grade gliomas. The optimal thresholds, as well as the corresponding sensitivities, specificities and accuracies, are summarized in Table 3. Table 4 presents results of statistical pairwise comparison of AUCs. Among all imaging parameters, SPF achieved the highest AUC value of 0.942, followed by *f* (0.896), D* (0.891) and K^trans^ (0.854), whereas the AUC values of diffusion indexes were relatively low (0.752 for ADC_0,1000_ and 0.732 for D, respectively). Further, the AUC of SPF was significantly higher than that of ADC_0,1000_ (*P* = 0.004). No significant difference in AUC values was detected between SPF and *f* (*P* = 0.317), as well as between SPF and K^trans^ (*P* = 0.065). By using SPF as a discriminative index, the diagnostic sensitivity, specificity and overall accuracy were 87.1% (27 of 31 high-grade gliomas), 94.7% (18 of 19 low-grade gliomas) and 90.0% (45 of all 50 patients), respectively, at the optimal cut-off value of 19.26%.

**Table 3:** Diagnostic performance of parameters for differentiation between low- and high-grade gliomas.

Parameter	Optimal threshold	Sensitivity (%)	Specificity (%)	Accuracy (%)
ADC_0,1000_ (×10^–^^3^ mm^2^/s)	≤1.252	83.9 (26/31)	57.9 (11/19)	74.0 (37/50)
SPF (%)	>19.26	87.1 (27/31)	94.7 (18/19)	90.0 (45/50)
*f* (%)	>5.81	90.3 (28/31)	78.9 (15/19)	86.0 (43/50)
D (×10^–^^3^ mm^2^/s)	≤1.217	83.9 (26/31)	57.9 (11/19)	74.0 (37/50)
D* (×10^–^^3^ mm^2^/s)	>10.514	87.1 (27/31)	84.2 (16/19)	86.0 (43/50)
K^trans^ (min^−1^)	>0.059	87.1 (27/31)	78.9 (15/19)	84.0 (42/50)
v_e_	>0.132	90.3 (28/31)	52.6 (10/19)	76.0 (38/50)
v_p_	>0.044	64.5 (20/31)	73.7 (14/19)	68.0 (34/50)

*Note—Data in parentheses are raw data*.

**Table 4:** *P* values by comparison of area under the curves (AUCs) for differentiation between low- and high-grade gliomas.

Parameter	ADC_0,1000_	SPF	*f*
SPF	0.004	…	…
*f*	0.086	0.317	…
K^trans^	0.200	0.065	0.485

*Note—Comparisons of AUC were performed with the DeLong method (DeLong et al., 1988). Adjusted level of significance was set at *P* = 0.008 with Bonferroni correction for six comparisons*.

## Discussion

In this study, we developed a simplified approach to obtain effective perfusion index from DWI by using only three *b*-values in human gliomas, and validated this technique against IVIM and DCE MR imaging modalities. The preliminary results showed that the perfusion-related parameter obtained from this 3-*b*-value approach, named SPF, correlated well with IVIM derived *f* and D*, and DCE MR imaging derived K^trans^. Furthermore, SPF proved to be useful in differentiating low- from high-grade gliomas, and showed higher diagnostic accuracy compared with ADC, IVIM and DCE MR imaging parameters. Thus, SPF may serve as a valuable alternative to measure tumor perfusion in gliomas in a noninvasive, convenient and efficient way.

One of the important histopathologic features associated with the grade of gliomas is microvascular proliferation, where high-grade gliomas are often characterized by exuberant vascularization compared with low-grade gliomas due to high tumor infiltrative nature. Thus, accurate assessment of neovascularization in gliomas is crucial for grading and further therapeutic decision making. In the field of medical imaging, DCE MR imaging and IVIM imaging are two importing imaging modalities to depict tissue perfusion information *in vivo*. In accordance with previous studies (Bisdas et al., 2013; Choi et al., 2013; Federau et al., 2014; Jung et al., 2014), our findings showed that DCE MR imaging derived K^trans^, v_e_ and v_p_, and IVIM derived *f* and D* were all significantly larger in high-grade gliomas than those in low-grade gliomas, indicating an increase in the perfusion of high-grade gliomas. Given that DW signal is affected by both water diffusion and microvascular perfusion, and the relative superiority depends on the chosen *b*-value, we derived SPF as the relative portion of the perfusion component (ADC_perf_) in the entire signal unit (ADC_0,200_). Conceptually, SPF has a physiologic function similar to *f* obtained from IVIM and v_p_ obtained from DCE MR imaging. Correlation analysis revealed a strong correlation between SPF and *f* (*ρ* = 0.732), and a weak correlation between SPF and v_p_ (*ρ* = 0.397). These distinct correlation results are in line with theoretical expectations: SPF and *f* associated tissue perfusion originates from the randomly orientated capillary network as the contribution from macrovascular structures is spoiled by applying the magnetic pulsed gradients (Le Bihan and Turner, 1992), whereas v_p_ measurement accounts for all types of vessels within the vascular bed regardless of vessel size (Sourbron and Buckley, 2011). Though a straightforward comparison between SPF and v_p_ has not been reported, a recently published study showed a positive correlation between *f* and v_p_ (*r* = 0.33) on untreated brain metastases (Kapadia et al., 2017), similar to our observations (*ρ* = 0.362). In addition, SPF also showed a strong correlation with D* (*ρ* = 0.716) and K^trans^ (*ρ* = 0.607). Based on these results, SPF proved to be a feasible perfusion metric comparable with quantitative IVIM and DCE MR imaging measurements. Further, our findings also added to existing evidence suggesting that there may exist an intrinsic association between perfusion indexes derived from contrast-free DWI and contrast-based DCE MR imaging, even though their mechanisms are fundamentally different.

Not surprisingly, we found that high-grade gliomas exhibited substantially elevated SPF values compared with low-grade gliomas. Further ROC analysis showed that SPF demonstrated the best diagnostic performance among all included parameters. Especially, a significantly higher AUC value for SPF was observed compared with ADC_0,1000_ or D (*P* < 0.008), indicating that perfusion features are more valuable for glioma grading. These results are consistent with previous studies revealing the added value of perfusion imaging to conventional DWI for a more accurate glioma grading (de Fatima Vasco Aragao et al., 2014; Togao et al., 2016). As ADC estimation with two *b*-values (e.g., ADC_0,1000_) is usually used in clinical routines, we suggest that an extra small *b*-value (e.g., 200 s/mm^2^) helps disentangle the perfusion information and the derived SPF adds value to ADC in the differentiation between low- and high-grade gliomas. Moreover, SPF showed a higher AUC value compared with *f*, though not reaching significance (*P* = 0.317), indicating that the reduction of the number of *b*-values did not necessarily result in the accuracy reduction. Besides the long examination time for IVIM, the widespread application of IVIM is limited for lack of standardized image acquisition and processing methods (Iima and Le Bihan, 2016). However, this simplified approach uses as few as three *b*-values and does not involve a complex algorithm. Thus, SPF may serve as a potentially time- and labor-saving alternative to IVIM without having to make compromise for diagnostic efficiency.

Of note, there is a technical issue related to SPF calculation. In the study, the high *b*-value of 1000 s/mm^2^ was used to to ensure that it was large enough so that the perfusion component is entirely vanished and thus only pure diffusion is preserved; meanwhile, it was yet small enough to guarantee a good signal-to-noise ratio and avoid kurtosis effect which contributes at high *b*-values (usually beyond 1000 s/mm^2^; Wu et al., 2015; While et al., 2017). We leveraged a cutoff *b*-value of 200 s/mm^2^ to separate pure diffusion component from the entire DW signal pool, which was empirically determined on the basis of some previous brain IVIM studies (Federau et al., 2012; Suh et al., 2014; Suo et al., 2016). Federau et al. (2012) and Suo et al. (2016) showed in their studies that a *b*-value of 200 s/mm^2^ might be considered to be an appropriate point to separate the perfusion decay and diffusion decay in the signal attenuation curve with a wide range of *b*-values. Use of different cutoff *b*-values may result in estimation bias. However, there is still no optimal cutoff *b*-value established for brain. Even so, our results showed a satisfying glioma grading performance with SPF using a cutoff *b*-value of 200 s/mm^2^. Future studies are still warranted to investigate the optimal cutoff *b*-value for SPF calculation in gliomas.

Another limitation of our study is that the number of patient population was relatively small. Our results need to be validated in a larger cohort and different glioma subtypes should be taken into account. In addition, our results could be specific to the hot spot ROI placement on solid portion of tumors. Whole-tumor segmentation with histogram analysis seems more reasonable to capture the heterogeneity of gliomas. Nevertheless, a recently published study comparing the different ROI delineation methods on ADC maps showed that hot spot method is clinically optimal for differentiating low- from high-grade gliomas compared with whole-volume histogram analysis in terms of time efficiency and diagnostic ability (Han et al., 2017).

In conclusion, the simplified approach to measure tissue perfusion based on DWI by using three *b*-values may be helpful to differentiate low- from high-grade gliomas. The reduced acquisition time, simple calculation method, and noninvasive nature of this approach may facilitate its clinical use, which can contribute to the more effective management of patients with gliomas.

## Author Contributions

MC and SS designed the experiments, performed the analysis and wrote the article. XH, KJ, YS, YW and WD performed the experiment and collected the data. JQ provided technical support for the magnetic resonance sequences. XZ and YZ contributed to the design of the experiment, image analysis and the manuscript revision.

## Conflict of Interest Statement

Author JQ is an employee of GE Healthcare China. The other authors declare that the research was conducted in the absence of any commercial or financial relationships that could be construed as a potential conflict of interest.
